# Pituitary Apoplexy: Description of Medical and Surgical Treatment and Clinical, Visual, and Endocrinological Outcomes During the SARS-CoV-2 Pandemic and Over Three Years

**DOI:** 10.7759/cureus.63152

**Published:** 2024-06-25

**Authors:** Luz M Pineda-Centeno, Ricardo A Palacios-Rodríguez, Tomas Moncada-Habib, Michel G Mondragon-Soto, Luis A Rodríguez-Hernández, Rodolfo Villalobos-Díaz, Victor Alcocer Barradas, Lesly A Portocarrero-Ortiz

**Affiliations:** 1 Neurology, National Institute of Neurology and Neurosurgery "Manuel Velasco Suarez", Mexico City, MEX; 2 Neurosurgery, National Institute of Neurology and Neurosurgery "Manuel Velasco Suarez", Mexico City, MEX; 3 Neuroendocrinology, National Institute of Neurology and Neurosurgery "Manuel Velasco Suarez", Mexico City, MEX

**Keywords:** outcome, sars-cov-2 pandemic, surgical treatment, medical treatment, pituitary apoplexy, pituitary center of excellence

## Abstract

Introduction: Pituitary apoplexy (PA) is a rare neuroendocrinological emergency. The SARS-CoV-2 pandemic recommendations led to a shift in the management of patients with pituitary diseases, especially in the decision-making between conservative and surgical treatment of patients with PA.

Objective: This study aimed to describe the conservative and surgical treatment and the clinical, visual, and endocrinological outcomes in patients with PA at the Pituitary Center of Excellence (PTCEO) during the SARS-CoV-2 pandemic and within three years.

Methods: This is a cohort study. Patients with PA between April 2020 and September 2023 were followed up. Treatment decisions, clinical manifestations, hormonal profile, and tumor size with MRI were described at the onset, at three months, six months, one year, two years, and three years after diagnosis.

Results: A total of 27 patients with PA diagnosis were included in the study. Of these, 12 patients were conservatively treated, six (50%) had prolactinomas, five (41.6%) had non-functioning adenomas, and one (8.3%) had pituicytoma. Fifteen patients were surgically intervened during the first hospitalization, nine (60%) had non-functioning adenomas, four (26.6%) had prolactinomas, one (6.6%) had ACTH-producing adenoma, and one (6.6%) had gonadotropinoma. Two patients from the conservatively treated group (one non-functioning adenoma and one pituicytoma) were intervened surgically at years 2 and 3, respectively.

During the initial assessment, there were no statistically significant differences between patients in visual acuity (9 [75%] vs 15 [100%]), visual field affection (8 [66.6%] vs 11 [73.3%]), and cranial nerve deficit (3 [25%] vs 6 [40%]). At six months follow-up, no statistically significant differences were found in the visual acuity improvement (8 [88%] vs 11 [100%]), visual field (8 [100%] vs 8 [72%]), and cranial nerve deficit between the two groups (3 [100%] vs 6 [100%]). Meanwhile, the average length of in-hospital stay was 1.5 vs 10 days (p = 0.019). The tumor size and largest diameter were smaller in the surgically treated group (6.2 vs. 0.5 cm^3^, p = 0.029 and 2.5 vs. 1.1 cm, p = 0.036, respectively). Visual acuity improved in nine (58.3%) patients at year 1: two (40%) conservative vs seven (100%) surgical (p = 0.039); six (85.7%) patients at year 2: two (66.6%) conservative vs. four (100%) surgical; and three (100%) patients on both groups at year 3. Fourteen patients needed hormonal substitution: 87.5% (eight [88.8%] conservative vs six [85.7%] surgical) at year 1, 85.7% (six patients in both groups) at year 2, and 80% (four conservative vs three [100%] surgical) at year 3. The thyrotropic axis was the most affected in both groups during the three years. During the first-year follow-up, six (85%) patients persisted with tumoral regression (2 [66.6%] conservative vs 4 [100%] surgical) and one (14.2%) patient from the medical group progressed. During the second and third years, 10 and three (100%) of the patients, respectively, showed the regression of the tumoral volume in both groups.

Conclusions: The clinical, visual, and neuroendocrinological outcomes were similar in both groups of patients with PA during the SARS-CoV-2 pandemic. In cases where the Pituitary Apoplexy Score (PAS) score does not surpass three points without neurological deterioration, conservative management can be considered an adequate option for treatment.

## Introduction

Pituitary apoplexy (PA) constitutes a neuro-endocrinologic emergency due to infarction, hemorrhage, or necrosis of a pituitary adenoma. The clinical manifestations are characterized by sudden severe headache, meningeal irritation, altered mental status, and visual deterioration [1,2]. It is a rare disease, affecting 2%-7% of all pituitary adenomas, and occurs mainly in non-functioning adenomas. In some cases, the PA can be the first manifestation of a pituitary adenoma [3,4]. It presents in the fifth and sixth decades of life in most cases, but it can manifest at any age [5,6].

Some of the described risk factors are a history of head trauma, arterial hypertension, use of anticoagulation therapy, major surgery, dynamic hypophyseal hormone tests (thyrotropin-releasing hormone [TRH], growth hormone-releasing hormone [GnRH], and corticotropin-releasing hormone [CRH]), dopaminergic agonists, and radiation [7].

There are no randomized clinical trials about the optimal management of PA. The consensus criteria for conservative management are non-neuroophthalmological compromise or only mild manifestations, while early surgical treatment (<7 days) in patients with severe impairment of visual acuity compromises of ocular movements and altered mental status [8,9]. The UK management guide suggests using the Pituitary Apoplexy Score (PAS) scale of 4 or more for urgent surgery [10,11].

In the literature, the difference in the impact between surgical and conservative management in the visual, cranial nerve, and endocrinologic outcome of patients with PA has not been well established [3,8,12,13]. Some studies have shown the benefit of surgical treatment in improving the visual deficit, and it has been recommended in patients with bitemporal hemianopia or amaurosis. The benefit of conservative management has been reported in selected cases [8,10,14-17].

During the SARS-CoV-2 pandemic, different medical societies have proposed recommendations for assessing and treating pituitary pathologies. The Pituitary Society suggested the stratification of emergent, urgent, and elective cases, and in some instances, even the transcranial approach was proposed as the method of choice [1]. When the SARS-CoV-2 pandemic emerged, there was not enough information about disease transmission in endoscopic endonasal surgery. Therefore, in this protocol, the surgery on non-urgent cases was postponed.

COVID-19 has been associated with various endocrine abnormalities, including disturbances in the hypothalamic-pituitary-adrenal (HPA) axis and thyroid dysfunction. Understanding these effects is crucial for managing patients with COVID-19 and potentially mitigating long-term complications. In this study, we determined the clinical, visual, and endocrinological outcomes in PA patients in both conservative and surgical treatment groups during the SARS-CoV-2 pandemic and within three years at a Pituitary Tumor Center of Excellence (National Institute of Neurology and Neurosurgery, Mexico City).

## Materials and methods

This is a cohort study that included patients with a diagnosis of PA between April 2020 and September 2023. The PA diagnosis was defined by clinical characteristics as severe acute headache, meningism symptoms, altered mental status, decreased visual acuity, campimetry deficit, and III, IV, V, and VI cranial nerve affection. MRI imaging with acute bleeding pituitary tumor was also needed. Tumoral size was assessed by the ellipsoidal formula. Patients’ medical records were revised for demographical information as well as medical history, clinical manifestations, and PAS.

Laboratory tests were performed including serum electrolytes, free tyrosine, TSH, cortisol, growth hormone, IGF-1, testosterone, LH, FSH, estrogens, and prolactin. Hypopituitarism was defined as the deficiency of three or more hormonal axes (gonadotrophic, tirotrophic, corticotropic, or somatotrophic), and panhypopituitarism was diagnosed if diabetes insipidus was also manifested.

After neuroendocrinological (biochemical) and neurologic exploration, the patients were assigned to one of the groups for treatment. The patients with PA and SARS-CoV-2 infection were conservatively treated. Patients with SARS-CoV-2-negative tests with non-neurological deficits or only mild visual symptoms were treated with the conservative approach. Surgical treatment was considered only for patients with severe visual symptoms (visual acuity 20/50 or worse) or neurological deterioration. The treatment modality was decided by the neuroendocrinology department.

Conservative treatment consisted of intravenous administration of dexamethasone 4 mg every eight hours + 100 mg of intravenous hydrocortisone initial dose followed by 50 mg every eight hours. This treatment scheme considered the peritumoral vasogenic anti-edema effect of dexamethasone, and due to its low mineralocorticoid effect, hydrocortisone was added at a substitutive dose because of the acute hypocortisolism. The surgical treatment group was prescribed an initial dose of 100 mg intravenous hydrocortisone, followed by 50 mg every eight hours. Ophthalmologic clinical examination, pituitary hormonal axis evaluation, and tumoral volume were assessed during the in-hospital stay, at seven days, one, three, and six months after diagnosis. Additionally, the pituitary hormonal axis was evaluated in 14, 13, and three patients at years 1, 2, and 3, respectively. Fifteen, nine, and three patients were followed up by the neuro-ophthalmology department at years 1, 2, and 3, respectively, and tumoral volume was calculated with MRI in nine, 12, and four patients at years 1, 2, and 3, respectively.

Qualitative variables were presented as frequencies and percentages. Quantitative variables were represented as means with standard deviation or median with interquartile ranges, depending on their distribution, which was previously determined with the Shapiro-Wilks test. Qualitative variables were compared with the Chi-square test or Fisher’s exact test. Quantitative variables were analyzed with the student’s t-test or the Mann-Whitney U test. Statistical significance was determined with a p < 0.05. SPSS version 24.0 (IBM Corp., Armonk, NY) statistical package was used.

## Results

A total of 27 patients were included. Twelve of them were treated with conservative management, while 15 patients were treated surgically. Two patients from the conservatively treated group (one with non-functioning adenoma and one with pituicytoma) were intervened surgically at years 2 and 3, respectively; therefore, they were included in the conservative management group during the follow-up. The mean age at the moment of diagnosis of PA was 47 and 38 years, respectively. Most of the patients were male in both groups (7 [58%] vs 10 [66.6%]). In the conservative management group, four patients were diagnosed with SARS-CoV-2 infection during the PA diagnosis, and one case was diagnosed two weeks before admission. In the surgical treatment group, one patient got COVID-19 five days after the surgery and died. Pituitary adenoma diagnosis was unknown until PA assessment in 12 (44%) patients: four (26.6%) in the surgical vs eight (66%) in the conservative groups, respectively.

In the conservative management group, six patients were diagnosed with prolactinoma, five with non-functioning adenoma, and one with pituicytoma. In the surgically treated group, nine had non-functioning adenomas, four had prolactinomas, one had ACTH-producing tumors, and one had gonadotropinoma. Differences in the visual deficit, campimetry defects, ptosis, and the affection of the endocrinological axis are described in Table 1. Tumor diameter was similar at admission in both groups (3.6 vs 3.9, p = 0.49) (Table 2).

**Table 1:** Clinical characteristics of the patients with PA at admission ^a^ Values are presented as mean and standard deviation. ^b^ Frequency and percentage. ^c^ Values are presented as mean and interquartile ranges 25 and 75. The Mann-Whitney U independent test was performed to determine the p-values. PA: Pituitary apoplexy; CN: Cranial nerves.

Variables	Conservative (n = 12)	Surgical (n = 15)	p
Age (years)^a^	47 ± 13	38 ± 12	0.061
Gender^b^ [Male n (%)]	7 (58.3)	10 (66.6)	0.70
Smoking, n (%)^b^	5 (41.6)	6 (40)	1.00
Alcoholism, n (%)^b ^	4 (33.3)	2 (13.3)	0.35
Diabetes mellitus, n (%)^b^	3 (25)	3 (20)	1.0
Arterial Hypertension, n (%)^b^	1 (8.3)	3 (20)	0.60
History of COVID-19, n (%)^b^	5 (41.6)	1 (6.6)	0.06
PA debut, n (%)^b^	8 (66.6)	4 (26.6)	0.05
Type of adenoma^c^			
Non-functioning adenoma	6 (50)	9 (60)	0.73
Prolactinoma	6 (50)	4 (26.6)	0.73
ACTH-producing	0	1 (6.6)	0.73
Gonadotropinoma	0	1 (6.6)	0.73
Pituitary Apoplexy Score (PAS)			
Total PAS (mean)^a^	2.67	2.93	0.58
Glasgow Coma Scale	15 (14-15)	15 (12-j15)	0.86
Visual acuity deficit, n (%)	9 (75)	15 (100)	0.07
Bilateral visual deficit, n (%)^b^	8 (66.6)	10 (66.6)	0.06
Visual field deficit, n (%)^c^	8 (66.6)	11(73.3)	0.89
Bitemporal hemianopia	4 (33.3)	6 (40)	0.89
Temporal hemianopia + amaurosis	3 (25)	4 (26.6)	0.89
Bilateral amaurosis	1 (8.3)	1 (6.6)	0.89
Cranial nerve deficit (third CN palsy), n (%)^b^	3 (25)	6 (40)	0.68
PAS, n (%)^b^			
<4	8 (66.6)	11 (73.3)	1.00
≥4	4 (33.3)	4 (26.6)	1.00

**Table 2:** Endocrinological profile and tumor size at the time of admission ^a^ Values are presented as mean and standard deviation. ^b^ Frequency and percentage.

Variables	Conservative (n = 12)	Surgical (n = 15)	p
Panhypopituitarism, n (%)^a^	1 (8.3)	1 (6.6)	1.00
Hypothyroidism, n (%)^a^	5 (41.6)	9 (60)	0.44
Hypocortisolism, n (%)^a^	2/7 (28.5)	3/6 (50)	1.0
Hypogonadism,n (%)^a^	4 (33.3)	8 (53.3)	0.67
Hyperprolactinemia,n (%)^a^	8 (66.6)	4 (26.6)	0.05
Tumoral volume, n (%)^b ^< 4 cm^3^	7 (58.3)	7 (46.6)	0.70
Tumoral volume, n (%)^b ^> 4 cm^3^	5 (41.6)	8 (53.3)	0.70

During the initial assessment, patients with conservative and surgical treatment had non-statistically significant differences in visual acuity (9 [75%] vs 15 [100%]), visual field affection (8 [66.6%] vs 11 [73.3%]), and cranial nerve deficit (3 [25%] vs 6 [40%]). At six months follow-up, there were no statistically significant differences in the visual acuity, visual fields, and cranial nerve deficit between the two groups. Meanwhile, the tumor size and largest diameter were smaller in the surgically treated group (6.2 vs 0.5 cm^3^, p = 0.029 and 2.5 vs 1.1 cm, p = 0.036, respectively) (Table 3).

**Table 3:** Clinical outcome and tumoral size according to the Pituitary Apoplexy Score in both groups of treatment at the time of admission and after six months of follow-up ^a^ Values are presented in frequency and percentage. X^2^ test was performed to determine the p-values. Vf: Visual fields; CN: Cranial nerves.

PAS components	Conservative (n = 12)	Surgical (n = 15)	p
Improvement in visual acuity, n (%)^a^	8/9 (88)	11/11 (100)	0.67
Complete improvement in visual acuity, n (%)^a^	3 (37.5)	4 (36)	0.67
Partial improvement in visual acuity, n (%)^a^	4 (50)	7 (64)	0.67
Deficit in visual acuity, n (%)^a^	1 (12.5)	0	0.67
Improvement in VF, n (%)^a^	8/8 (100)	8/11 (72)	0.62
Complete improvement in VF, n (%)^a^	2 (25)	3 (37)	0.62
Partial improvement in VF, n (%)^a^	5 (62)	5 (63)	0.62
Deficit in VF, n (%)^a^	1 (13)	0	0.62
Improvement in CN deficit, n (%)^a^	3/3 (100)	6/6 (100)	0.55
Complete improvement in CN deficit, n (%)^a^	3 (100)	5 (83)	0.55
Partial improvement in CN deficit, n (%)^a^	0	1 (17)	0.55
Volume at admission, cm^3^	9.3 (4.2-33)	20.9 (10.2-33.8)	0.32
Largest diameter at admission, cm^d^	3.6 +1.4	3.9 + 1.0	0.49
Volume at 6 months, cm^3^	6.2 (1.2-10.5)	0.50 (0-3.7)	0.029
Largest diameter at 6 months, cm^d^	2.5 (1.5-3.2)	1.1 (0-2.3)	0.036

Visual acuity improved (p = 0.039) in nine (58.3%) patients at year 1: two (40%) conservative vs seven (100%) surgical; six (85.7%) patients at year 2: two (66.6%) conservative vs four (100%) surgical; and three (100%) patients on both groups at year 3. One patient (20%) from the medical group had worsened visual acuity at year 1. Visual field deficits improved in four patients (66.6%): four (100%) and one in the conservative group vs two in the surgical group (100%) at years 1, 2, and 3, respectively. The visual acuity at year 1 was the only statistically significant difference within three years, with a p-value of 0.039. The results are shown in Tables 4, 5.

**Table 4:** Visual acuity outcomes at years 1, 2, and 3 ^a ^Values are presented in frequency and percentage. The Fisher’s exact test was used to determine the p-values.

Variables	Conservative	Surgical	p
Year 1 follow-up^a^	n = 5	n = 7	
Improved, n (%)	2/5 (40)	7/7 (100)	0.039
Stable, n (%)	2/5 (40)	0/7 (0)	
Worsens, n (%)	1/5 (20)	0/7 (0)	
Year 2 follow-up^a^	n = 3	n = 4	
Improved, n (%)	2/3 (66.6)	4/4 (100)	0.46
Stable, n (%)	1/3 (33.3)	0/4 (0)	
Worsens, n (%)	0/3 (0)	0/4 (0)	
Year 3 follow-up^a^	n = 1	n = 2	
Improved, n (%)	0/1 (0)	2/2 (100)	0.33
Stable, n (%)	1/1 (100)	0/2 (0)	
Worsens, n (%)	0/1 (0)	0/2 (0)	

**Table 5:** Visual field outcomes at years 1, 2, and 3 ^a^ Values are presented in frequency and percentage. The Fisher’s exact test was used to determine the p-values.

Variables	Conservative	Surgical	p
Year 1 follow-up^a^	n = 6	n = 6	
Improved, n (%)	4/6 (66.6)	4/6 (66.6)	1.0
Stable, n (%)	1/6 (16.6)	2/6 (33.3)	
Worsens, n (%)	1/6 (16.6)	0/6 (0)	
Year 2 follow-up^a^	n = 4	n = 4	
Improved, n (%)	4/4 (100)	4/4 (100)	1.0
Stable, n (%)	0/4 (0)	0/4 (0)	
Worsens, n (%)	0/4 (0)	0/4 (25)	
Year 3 follow-up^a^	n = 1	n = 2	
Improved, n (%)	1/1 (100)	2/2 (100)	0.58
Stable, n (%)	0/1 (0)	0/2 (0)	
Worsens, n (%)	0/1 (0)	0/2 (0)	

At one-month follow-up, there was a significant difference in hypocortisolism in the surgically treated patients (0 = 0% vs 4 = 100%, p < 0.001), whereas the hyperprolactinemia was significantly higher in the conservatively treated group (3 = 43% vs 0 = 0%, p = 0.05). There were no significant differences between both groups respecting other endocrinological abnormalities. At six months follow-up, there were no statistically significant differences between both groups in the endocrinological profile (Table 6).

**Table 6:** Endocrinological outcomes and complications in both groups of treatment at one and six months ^a ^Values are presented in frequency and percentage. The Fisher’s exact test was used to determine the p-values. CSF: Cerebrospinal fluid.

Variables	Conservative (n = 12)	Surgical (n = 15)	p
Deceased, n (%)^a^	0 (0)	4 (26.6)	0.10
In-hospital days	1.5 (0-12.25)	10 (5-21)	0.019
Complications, n (%)^a^			
Meningitis	1 (8.3)	1 (6.6)	1.00
CSF fistula	0 (0)	5 (33.3)	0.18
Diabetes insipidus	0	10 (66.6)	<0.01
One-month follow-up^a^	n = 11	n = 9	
Diabetes insipidus, n (%)	2 (16.6)	4 (26.6)	0.66
Hypocortisolism, n (%)	0/6 (0)	4/4 (100)	0.004
Hypothyroidism, n (%)	5/6 (83)	5/5 (100)	0.43
Hypogonadism, n (%)	1/6 (16)	4/4 (100)	0.18
Hyperprolactinemia, n (%)	3/7 (43)	0/3 (0)	0.05
Hypoprolactinemia, n (%)	1/7 (14)	1/3 (33)	0.10
Hypopituitarism, n (%)	0/6 (0)	4/4 (100)	0.10
Panhypopituitarism, n (%)	1/6 (0)	1/4 (25)	0.30
Six-month follow-up	n = 11	n = 9	
Diabetes insipidus, n (%)	0/9 (0)	3/8 (38)	0.21
Hypercortisolism, n (%)	3/8 (38)	4/7 (57)	0.61
Hypothyroidism, n (%)	8/10 (80)	5/7 (71)	1.00
Hypogonadism, n (%)	3/6 (50)	6/7 (87)	0.26
Hyperprolactinemia, n (%)	2/9 (22)	2/7 (29)	1.00
Hypoprolactinemia, n (%)	2/9 (22)	2/7 (29)	1.00
Hypopituitarism, n (%)	3/6 (50)	4/7 (57)	0.26
Panhypopituitarism, n (%)	0 (0)	1 (14)	0.44

Patients needed hormonal substitution in 87.5% (8 = 88.8% conservative vs 6 = 85.7% surgical) at year 1, 85.7% (six patients in both groups) at year 2, and 80% (four conservative) vs 100% (three surgical) at year 3. The thyrotropic axis was the most affected in both groups during the three years (8 [80% conservative] vs 6 [85.7% surgical]; 85.7% [six patients from each group]; and 75% [3 patients from each group]) at years 1, 2, and 3, respectively. This was followed by the corticotropic axis with 70.5% (12 patients in total): six (60%) conservative vs six (85.7%) surgical at year 1; 78.5% (12 patients in total​): seven (100%) conservative vs five (71.4%) surgical at year 2; and 75% (three patients from each group) deficit at year 3. Only two patients from the medical group needed testosterone replacement during the first and second years (11.7% and 14.2%), and one patient from the conservative group needed it during the third year (12.5%). One patient from the surgical group needed desmopressin during the three years, and another patient from the surgical group estrogen/progesterone replacement needed it during the same period (5.8%, 7.1%, and 12.5%). The results are shown in Tables 7, 8.

**Table 7:** Hormonal replacement outcomes at years 1, 2, and 3 ^a ^Values are presented in frequency and percentage. The Fisher’s exact test was used to determine the p-values.

Variables	Conservative	Surgical	p
Year 1 follow-up^a^	n = 9	n = 7	0.72
Hormonal replacement, n (%)	8/9 (88.8)	6/7 (85.7)	
No hormonal replacement, n (%)	1/9 (11.1)	1/7 (14.2)	
Year 2 follow-up^a^	n = 7	n = 7	1.0
Hormonal replacement, n (%)	6/7 (85.7)	6/7 (85.7)	
No hormonal replacement, n (%)	1/7 (14.2)	1/7 (14.2)	
Year 3 follow-up^a^	n = 4	n = 4	1.0
Hormonal replacement, n (%)	4/5 (80)	3/3 (100)	
No hormonal replacement, n (%)	1/5 (20)	0/3 (0)	

**Table 8:** Hormonal replacement therapy outcomes at years 1, 2, and 3 ^a ^Values are presented in frequency and percentage. The Fisher’s exact test was used to determine the p-values.

Variables	Conservative	Surgical
Year 1 follow-up^a^	n = 10	n = 7
Levothyroxine, n (%)	8/10 (80)	6/7 (85.7)
Prednisone, n (%)	6/10 (60)	6/7 (85.7)
Cabergoline, n (%)	6/10 (60)	1/7 (14.2)
Testosterone, n (%)	2/10 (20)	0/7 (0)
Desmopressin, n (%)	0/10 (0)	1/7 (14.2)
Estrogen/progesterone, n (%)	0/10 (0)	1/7 (14.2)
Year 2 follow-up^a^	n = 7	n = 7
Levothyroxine, n (%)	6/7 (85.7)	6/7 (85.7)
Prednisone, n (%)	7/7 (100)	5/7 (71.4)
Cabergoline, n (%)	6/7 (85.7)	1/7 (14.2)
Testosterone, n (%)	2/7 (28.5)	0/7 (0)
Desmopressin, n (%)	0/7 (0)	1/7 (14.2)
Estrogen/progesterone, n (%)	0/7 (0)	1/7 (14.2)
Year 3 follow-up^a^	n = 4	n = 4
Levothyroxine, n (%)	3/4 (75)	3/4 (75)
Prednisone, n (%)	3/4 (75)	3/4 (75)
Cabergoline, n (%)	3/4 (75)	0/2 (0)
Testosterone, n (%)	1/4 (25)	0/4 (0)
Desmopressin, n (%)	0/4 (0)	1/4 (25)
Estrogen/progesterone, n (%)	0/4 (0)	1/4 (25)

Tumoral volume size at admission in the conservative group was 9.3 cm^3^ (4.22-33) and 20.9 cm^3^ (10.2-33.8) in the surgically treated patients. The largest diameter was 3.6 (SD 1.4) and 3.9 (SD 1.0), respectively. At six months follow-up, the final tumoral size was 6.2 cm^3^ (1.2-10.5) with the largest diameter of 2.5 (1.5-3.2) in the conservative group and 0.50 (0-3.7) with the largest diameter of 1.1 (0-2.3) in the surgical group with statistically significant differences of 0.029 and 0.036, respectively. In this study, we considered a change of ±10% in the tumoral volume to define progression or regression during the next three years. During the first-year follow-up, six (85%) patients persisted with tumoral regression: two (66.6%) conservative vs four (100%) surgical, and one (14.2%) patient from the medical group progressed. During the second and third years, 10 and three (100%) of the patients showed the regression of the tumoral volume in both groups. The results are shown in Table 9.

**Table 9:** Tumoral volumes at years 1, 2, and 3 ^a ^Values are presented in frequency and percentage. The Fisher’s exact test was used to determine the p-values.

Variables	Conservative	Surgical	p
Year 1 follow-up^a^	n = 3	n = 4	0.46
Progression, n (%)	1/3 (33.3)	0/4 (0)	
Stable, n (%)	0/3 (0)	0/4 (0)	
Regression, n (%)	2/3 (66.6)	4/4 (100)	
Year 2 follow-up^a^	n = 5	n = 6	1.0
Progression, n (%)	0/5 (0)	0/6 (0)	
Stable, n (%)	0/5 (0)	0/6 (0)	
Regression, n (%)	5/5 (100)	5/5 (100)	
Year 3 follow-up^a^	n = 1	n = 2	0.58
Progression, n (%)	0/1 (0)	0/2 (0)	
Stable, n (%)	0/1 (0)	0/2 (0)	
Regression, n (%)	1/1 (100)	2/2 (100)	

Complications were more frequent in the surgically treated group, with diabetes insipidus occurring in 10 (66.6% surgical) vs 0 (0% conservative) patients (p < 0.01), CSF fistula in five (33.3% surgical) vs 0 (0% conservative) patients. The mortality was 26% (four patients) in the surgical group (p = 0.10), and there was one post-surgical in-hospital COVID-19 pneumonia, one case of postoperative hemorrhage, one case of hydrocephalus, and one decease after discharge with a neurological acute deficit, where the family decided not to seek medical attention. During the first-year follow-up, one patient from the surgically treated group died from acute renal and respiratory failure, and one patient from the conservative group died from myocardial infarction.

## Discussion

In the latest systematic reviews, which comprehend the last 30 years, the most affected age group by PA was those over 50 years old (58.8 + 14.9 and 53.8 + 19.4 in the conservative vs surgical groups, respectively) [14]. The patients evaluated in our series are younger than the previously reported series, with a mean age of 47 years in the conservative group and 38 years in the surgical group. Similar to most reports, just over half of the patients were male [13,18].

We found that more than half of the patients debuted with PA with no previous pituitary adenoma diagnosis, which is similar to what has been previously described, where 57%-85% of the patients were unaware of their disease [13]. Nonetheless, only four (26%) of the surgically treated patients debuted with PA, with a statistically significant difference. This could be due to the size of the tumor and visual or neurological deficit with higher incidence in this group of patients than the conservative group.

According to the UK management guidelines for PA, surgical resection is recommended for patients with PAS of 4 or more. At the initial assessment, the percentage of patients with this score was lower. There were no significant differences between both groups, although some studies consider this score useful for determining the ideal intervention [9].

Some studies have demonstrated that surgical treatment has better results in those patients who present with acute or progressive visual acuity deficit, ophthalmoplegia, and altered mental status. Despite that, in some cases, the hormonal axis does not recover functionality [9,16,19].

Compared to the clinical review by Almeida et al. [14], we found that the diminishment in visual acuity is the most common clinical manifestation of PA, and it was more prevalent in our surgical group, comprehending 100% (15 patients) of the participants. It is important to mention that over half of the patients presented with bilateral visual deficits, which is one of the main indications for surgical treatment.

Marx et al. found that visual field deficit was present in 24% of the patients in the conservative group and 73% in the surgical group [13]. These values were close to the present studied series, where visual field deficit was 66% (eight patients) and 73% (11 patients), respectively. In both series, the main deficit was bitemporal hemianopia; both groups had one case of bilateral amaurosis.

Cortisol levels were not valid because the laboratory work was obtained heterogeneously after the initial substitution treatment with steroids in both groups (9/15, 60% in the surgical group, and 5/12, 41.6% in the conservative treatment group). This conduct was present because of the limited availability of laboratory studies during the pandemic. The thyrotrope axis had a similar behavior in both groups, affecting nearly half of the patients. Respecting the hormonal axis’s abnormalities at admission, in previous reports, more than 50% of cases had hypocortisolism at the time of diagnosis. Hypocortisolism was found only in 2/7 (28.5%) patients in the conservative group and 3/6 (50%) in the surgical group.

There is evidence of benefits in the hormonal axis’ recovery of both types of treatment [12,14,16,20]. Like in most series, our series did not show statistical differences regarding the type of management. The tumor volume and largest diameter were similar in both groups, but when compared to the series reported in the literature, the mean largest diameter was almost double in size (3.6 + 1.4 cm in the conservative group vs 3.9 + 1.0 cm in the surgical group) vs (2.2 cm in the conservative group vs 2.7 cm in the surgical group) [14]. Considering that the mean maximal diameter was similar in both groups, this was not a criterion for opting for surgical treatment.

During the follow-up, the tumoral size reduction, as expected, was significantly higher in the surgical treatment group. When compared at the six-month follow-up, the mean maximum diameter was also lower in the conservative group, decreasing from 3.2 to 2.4 cm. Some series reported that patients with conservative management have shown up to 82% of mass reduction [2,18] and, even in some cases, complete tumoral remission posterior to the PA event [19,21-23].

There was no significant difference in the visual acuity outcome between both groups, but they are slightly higher when compared to the percentages reported (85% conservative and 82% surgical). Nonetheless, complete visual acuity recovery was lower than the one reported in the literature for both groups [7].

Visual field outcomes were in the same pattern as the latest series, with improvement in more than two-thirds of the patients [3,8,16,24]. Our series reported 100% (eight patients) improvement in the conservative management group and 72% (eight patients) in the surgical management group, but only less than 1/3 of the patients in both groups achieved complete visual recovery.

The cranial nerve deficit recovery in both groups in our series was superior when compared with the latest meta-analysis (3 = 100% vs 5 = 83% and 85%) [25]. It is of significance that most patients in this series received medical management with high doses of dexamethasone, whose effect diminishes the edema of the ophthalmic nerve and the oculomotor nerves, presumably achieving a better clinical outcome for our patients.

From the endocrinological perspective, our series is comparable with the meta-analysis by Fleseriu [25] at one-month and six-month follow-ups. While there was endocrinological dysfunction in five cases in the surgical group, the conservative management group had none.

In recent years, it has been observed that conservative medical treatment in well-selected patients has shown favorable results in the outcome of the patients [13]. However, the treatment of choice for PA remains undetermined up to this moment due to the disagreement between the obtained results.

At our institution, we did not count on further experience in the conservative management of patients with PA. However, the SARS-CoV-2 pandemic and the new recommendations of management urged our healthcare team to find an alternative therapeutical option for patients with these characteristics.

With the widespread administration of COVID-19 vaccines, it is important to consider their potential effects on the pituitary gland. This could include autoimmune reactions, inflammatory responses, or other mechanisms that may affect hormonal regulation. Understanding the potential mechanisms of vaccine-induced PA has significant implications for vaccine safety monitoring and public health efforts [26,27].

Some authors have studied the link between pituitary dysfunction and persistent COVID-19 symptoms, involving disturbances in the hypothalamic-pituitary-adrenal (HPA) axis, alterations in hormone production and secretion, or interactions between the immune system and the endocrine system [27,28].

The limitations of our study include the selection criteria that were not established objectively and incomplete follow-ups after three years for most of the patients. We considered the number of patients treated conservatively as a strength as this was considered a neurosurgical emergency in our institution, allowing us to change the management.

## Conclusions

The clinical, visual, and neuroendocrinological outcomes are similar between both treatments in patients with PA during the initial clinical stages. During the SARS-CoV-2 pandemic, conservative management was considered the first line of treatment for selected patients. We can conclude that this paradigm can be of choice considering the evidence of improvement in our patients. The outcome regarding tumor volume is better in patients in the surgical treatment group, but we must consider that the medically managed patients had a volume diminishment after six months, one year, two years, and three years of follow-up. More studies with a rigorous methodology are needed to validate our findings.
